# Evaluation of deep learning methods for parotid gland segmentation from CT images

**DOI:** 10.1117/1.JMI.6.1.011005

**Published:** 2018-10-01

**Authors:** Annika Hänsch, Michael Schwier, Tobias Gass, Tomasz Morgas, Benjamin Haas, Volker Dicken, Hans Meine, Jan Klein, Horst K. Hahn

**Affiliations:** a Fraunhofer MEVIS, Bremen, Germany; b Varian Medical Systems Imaging Laboratory GmbH, Baden-Dättwil, Switzerland; c Varian Medical Systems, Las Vegas, Nevada, United States

**Keywords:** deep learning, segmentation, autocontouring, radiotherapy planning, head and neck

## Abstract

The segmentation of organs at risk is a crucial and time-consuming step in radiotherapy planning. Good automatic methods can significantly reduce the time clinicians have to spend on this task. Due to its variability in shape and low contrast to surrounding structures, segmenting the parotid gland is challenging. Motivated by the recent success of deep learning, we study the use of two-dimensional (2-D), 2-D ensemble, and three-dimensional (3-D) U-Nets for segmentation. The mean Dice similarity to ground truth is ∼0.83 for all three models. A patch-based approach for class balancing seems promising for false-positive reduction. The 2-D ensemble and 3-D U-Net are applied to the test data of the 2015 MICCAI challenge on head and neck autosegmentation. Both deep learning methods generalize well onto independent data (Dice 0.865 and 0.88) and are superior to a selection of model- and atlas-based methods with respect to the Dice coefficient. Since appropriate reference annotations are essential for training but often difficult and expensive to obtain, it is important to know how many samples are needed for training. We evaluate the performance after training with different-sized training sets and observe no significant increase in the Dice coefficient for more than 250 training cases.

## 1 Introduction

High radiation dose on organs at risk (OAR) during radiotherapy (RT) treatment can have severe side effects. An important set of OAR during treatment of head and neck cancer are the parotid glands, which are a type of salivary glands that are very sensitive to radiation. The most common radiation-induced side effect on the parotid glands is xerostomia (dry mouth), which can significantly decrease life quality.^1^ Therefore, modern radiotherapy planning requires accurate segmentation of target structures and OAR for precise and highly localized dose planning.^2^ As manual segmentation of the planning images is very time-consuming and user-dependent, radiotherapy planning could highly benefit from automatic methods for contouring. However, fully automatic segmentation of the parotid glands from computed tomography (CT) images is difficult due to their high variability in shape and appearance and often low soft-tissue contrast to surrounding structures. Because of their anatomical location, they are also prone to be affected by dental metal artifacts.

Various methods other than deep learning have been proposed for automatic segmentation of OAR in the head and neck region. The approaches include, among others, (multi) atlas-based methods,^3^^–^^6^ model-based methods,^7^^–^^10^ or their combinations.^11^^,^^12^ Some of the methods^6^^,^^8^^–^^11^ have been evaluated in the 2015 MICCAI challenge on head and neck autosegmentation, where the best mean Dice score on the parotid glands was 0.84.^13^

Since about 2012, deep learning methods have been extensively used for medical image processing problems, with a remarkable proportion of published papers on segmentation applications.^14^^,^^15^ Some of the most popular deep neural network architectures for segmentation include the U-Net,^16^^,^^17^ V-Net,^18^ and multiresolution architectures such as DeepMedic^19^ and F-Net.^20^ Some approaches have also been made with recurrent neural networks,^21^ generative adversarial networks,^22^ and other neural network architectures proposed by the computer vision community. Moreover, deep neural networks have proven to be powerful in several recent segmentation challenges such as brain tumor segmentation,^23^ liver tumor segmentation,^24^ and ischemic stroke lesion segmentation.^25^

Deep learning methods have also already been applied to various steps in the RT workflow, including but not limited to automatic contouring.^26^^,^^27^ In first clinical validations, deep learning segmentation methods have shown to decrease the total time required for OAR contouring in comparison to manual and atlas-based contouring.^28^ Head and neck segmentation from CT images has also been previously addressed using deep learning.^29^^,^^30^ However, while Ibragimov and Xing^30^ could observe superior performance on many OAR to previously reported results using their neural network, the performance on parotid gland segmentation only was comparable to previously reported Dice coefficients in the literature. Fritscher et al.^29^ also reported on the promising results of using deep learning methods for head and neck segmentation. All of these recent successes of deep learning in various medical domains motivate us to further explore deep learning for parotid gland segmentation from CT images.

In this contribution, we study parotid gland segmentation using deep learning, focusing on the U-Net architecture^16^^,^^17^ that we apply in two-dimensional (2-D), three-dimensional (3-D), and in a 2-D ensemble mode. We also evaluate the performance of the trained neural networks on the publicly available test dataset of the 2015 MICCAI challenge on head and neck autosegmentation and compare to the model and atlas-based methods that competed in the challenge. We show that the deep learning approaches yield superior results based on the obtained Dice coefficients, which are statistically significant for all but one compared method. Another important aspect of deep learning is the availability of a large, annotated training dataset that ideally captures most of the anatomical variability. In practice, medical datasets are often rather small compared to the typical datasets used in the computer vision community such as the ImageNet dataset.^31^ We investigate the influence of the number of training samples on the resulting Dice coefficients on a validation set.

## 2 Methods

### 2.1 Data

The image data used in the study consisted of 254 head and neck CT scans from two different clinical sites. For each CT scan, clinical routine level, uncurated reference segmentations of the left and right parotid glands, created by a single physician per image, were available. As the contours come from clinical routine, several doctors were involved in the contour creation at both clinical sites. The axial in-plane resolution was either 0.977 or 1.172 mm for all but two images, for which it was 1.219 mm. The slice spacing was either 2 or 3 mm. Due to the clinical uncurated nature of the contours, inconsistencies in the contouring of different scans are to be expected.

To simplify the segmentation problem, we decided to focus on a binary segmentation task (see Sec. 2.3). Therefore, all neural networks were trained to segment the left parotid gland only. The image data and reference segmentations of the right parotid gland were used for data augmentation by mirroring it and thus included into the training and validation. In total, 507 reference segmentations (253 left side, 254 right side mirrored, 1 left parotid gland was resected) were used and divided into 467 examples for training and 40 examples for validation.

In addition, for testing on an independent and publicly available test set, the 10 off-site and 5 on-site test cases of the 2015 MICCAI challenge on head and neck autosegmentation^13^ were used. The test data, including image data and carefully drawn segmentations based on best practices and scientific literature, are available at the Public Domain Database for Computational Anatomy.^32^

### 2.2 Preprocessing

The axial in-plane resolution of the training and validation data was close to $1\times 1\;{\mathrm{mm}}^{2}$ for all images but the slice spacing was either 2 mm or 3 mm. Therefore, in order to preserve details in the axial plane but unify the resolution along the transverse axis, the data were resampled along the transverse axis only to 2 mm slice spacing using a Lanczos kernel with support size 3. For inference, the independent MICCAI challenge test data were resampled to $1\times 1\times 2\;{\mathrm{mm}}^{3}$ to match the resolution used during training of the neural networks. Furthermore, the data were preprocessed by automatically removing the treatment couch via masking of the patient volume.

### 2.3 Neural Networks

**Fig. 1:**
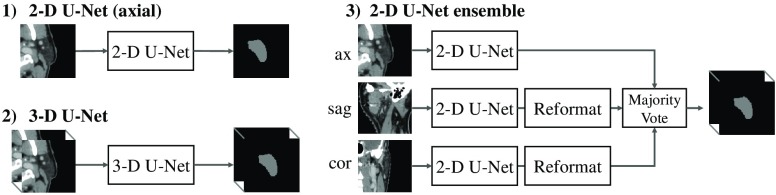
Schematic drawing of the different neural network architectures used: (1) 2-D U-Net working on 2-D patches, (2) 3-D U-Net working on 3-D patches, and (3) ensemble of three 2-D U-Nets working on axial, coronal, and sagittal patches, respectively. The individual predictions of the ensemble are combined to a single prediction via a majority vote.

**Fig. 2:**
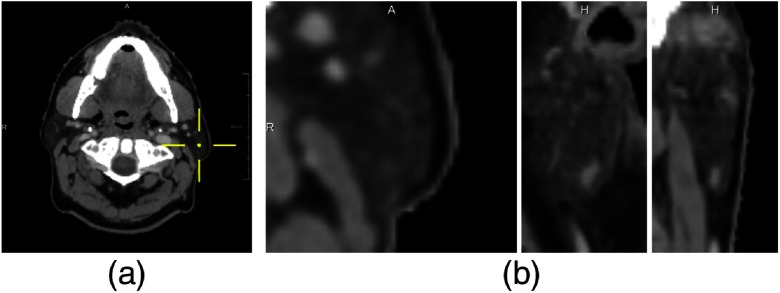
Receptive field visualization of a 3-D U-Net with three resolution levels: for the classification of the voxel marked with a cross hair in (a), the context that the neural network can use for classification is a cuboid of size 44×44×44 voxels or $44\times 44\times 88\;{\mathrm{mm}}^{3}$. The central planes through the cuboid in axial, sagittal and coronal view are visualized in (b).

We chose the U-Net architecture^16^^,^^17^ as the basis of all experiments. Three different U-Net models were implemented: a 2-D U-Net trained on axial slices, a 3-D U-Net, and a 2-D U-Net ensemble, as schematically shown in Fig. 1. The 2-D U-Net ensemble consisted of three 2-D U-Nets trained on axial, coronal, and sagittal reformatted slices, respectively, whose predictions were combined via a majority voting. This ensemble approach for combining orthogonal view directions is an alternative to 2.5-D networks, where multiple slices from orthogonal image planes are simultaneously fed into one neural network.^33^ All neural networks were trained to solve the binary segmentation task of segmenting the left parotid gland only. As a consequence, they had two output channels for foreground and background, followed by a softmax layer. All neural networks were implemented in-house in accordance with the architecture descriptions by Ronneberger et al.^16^ and Ciçek et al.,^17^ using the deep learning framework Lasagne.^34^ However, in contrast to the U-Net originally described in Ref. 16, batch normalization^35^ was also used for the 2-D U-Nets and only three of the U-Net’s internal resolution levels were implemented. The resulting U-Net has a maximum receptive field size of 44×44 voxels in the 2-D case and 44×44×44 voxels in the 3-D case, which contribute to the classification of a single voxel. For an image resolution of $1\times 1\times 2\;{\mathrm{mm}}^{3}$, this corresponds to a receptive field size of $44\times 44\times 88\;{\mathrm{mm}}^{3}$, as visualized in Fig. 2. The receptive field depends on the order and number of convolutional and pooling layers and their kernel sizes and should not be confused with the patch size used for training.^36^ From Fig. 2, the receptive field size seems sufficient as the parotid gland is almost completely inside the receptive field for a voxel chosen at the gland’s center. Moreover, the resulting U-Nets have fewer parameters than a U-Net with more resolution levels, hence they are easier to train on a GPU with limited memory.

### 2.4 Training

All training of neural networks was performed on a desktop computer with an NVIDIA GeForce GTX 1080 graphics card with 8 GB graphical memory. Batch sizes, number of epochs, and learning rates were adapted to the memory consumption and training convergence of each individual neural network.

As the structure to be segmented is small compared to the full volume, a strategy for increasing the foreground percentage during training is necessary. Otherwise, the network will mostly see background and not learn the segmentation task. The problem is addressed to some extent by using a soft Dice loss for training of all U-Nets, which accounts for low foreground percentage during the loss calculation.^18^ Furthermore, two different strategies to actually increase the foreground percentage during training were applied for the 2-D and 3-D U-Nets, respectively.

#### 2.4.1 Training in a region of interest

For the 2-D U-Nets, a region of interest (ROI)-based training strategy was applied. During training, slices to be presented to the neural network were drawn only from within the ROI. It was defined by any slices (axial, sagittal, or coronal reformatted depending on the view direction of the neural network) containing the left or mirrored right parotid gland. Additionally, the U-Net should learn not to segment anything on slices directly neighboring the organ. Therefore, an additional margin of five slices in view direction was included into the ROI. As the scan range of head and neck scans can be large, a margin of 25 voxels above and below the reference segmentation was additionally applied to sagittal and coronal reformatted slices in order to further increase the foreground percentage.

#### 2.4.2 Patch-based training with controlled batch composition

For the 3-D U-Net, the ROI training strategy could not be applied as the 3-D ROI did not fit into GPU memory together with the neural network. Therefore, a patch-based training with controlled batch composition was implemented. Patches of size 72×72×56 voxels were extracted from the full volume instead of an ROI. From these patches, the center 32×32×16 voxels were classified and the remainder was padding, which was needed for the U-Net’s specific architecture with unpadded convolutions, where the image size shrinks with each convolutional layer. The padding also serves to provide context for the output neurons toward the borders of the unpadded patch. Class balancing of foreground and background was performed by composing each minibatch during training so that 50% of the patches overlapped with the parotid gland.

In addition to training on the full training dataset with 467 cases, the 2-D and 3-D U-Nets were trained on subsets of 50, 150, 250, 350, and 450 training cases in order to investigate the impact of training data quantity on the segmentation results.

### 2.5 Postprocessing

The U-Nets’ predictions were binarized by thresholding at 0.5. A connected component (CC) analysis was performed and the largest component was taken as the final segmentation result with the aim to eliminate small false-positive findings. In our application, this basic postprocessing seems to be sufficient, as we expect to find a single left parotid gland inside each image volume as the largest segmented structure. Furthermore, the use of the Dice loss for training produces a raw neural network output, which is already close to binary with values either very close to 0 or 1. Therefore, no complex algorithm for converting soft predictions to hard labels is necessary in this case. In more complex detection tasks such as brain lesion segmentation, where the number of structures to be detected is unknown and prediction maps may be soft, additional advanced postprocessing using conditional random fields or other machine learning algorithms can be necessary.^19^

Finally, all segmentation results were resampled to the original image resolution using nearest-neighbor interpolation, in order to be able to compare to the original reference contours.

### 2.6 Evaluation

The quantitative evaluation was mainly based on the Dice coefficient.^37^ For two sets of voxels X and Y, which represent segmentation result and reference segmentation, it is given by

$$\mathrm{DSC}(X,Y)=\frac{2|X\cap Y|}{\left|X|+|Y\right|}.$$ (1)

To evaluate the overall over- versus underestimation in Sec. 3.2, we further compute an average signed surface distance (ASSD) integrated into the development platform MeVisLab.^38^ For sets of voxels X and Y, it is given as

$$\mathrm{ASSD}(X,Y)=\frac{1}{\left|\partial X|+|\partial Y\right|}[\underset{x\in \partial X}{\sum }d(x,Y)-\underset{y\in \partial Y}{\sum }d(y,X)],$$ (2)

where |∂X| denotes the border voxels of X and

$$d(x,Y)=\{\begin{array}{ll} \inf_{y\in \partial Y}{\left\|x-y\right\|}_{2}, & \text{if}\;x\in Y^{C}\\ -\inf_{y\in \partial Y}{\left\|x-y\right\|}_{2}, & \text{if}\;x\in Y \end{array},$$ (3)

is the signed Euclidean distance of a voxel x to the set Y. The distance measure is derived from the average or mean surface distance often used for segmentation evaluation and challenges.^13^^,^^39^ However, instead of calculating absolute differences, signed distances are used. This way, the distance measure is positive outside of the reference mask and negative inside it, therefore it can be used to distinguish over- and underestimation. For statistical evaluation, we used the Wilcoxon-signed rank test at significance level 0.05 to compare the performance of different neural networks.

## 3 Results

The results are presented in four parts. First, the 2-D U-Net, 2-D U-Net ensemble, and 3-D U-Net are evaluated on the validation data and the effect of the training strategies is described. Second, the performance of the 2-D ensemble and 3-D U-Net on validation versus MICCAI test data is examined. Then, the deep learning results are compared to the quantitative results of the 2015 MICCAI challenge. Finally, the impact of the number of training samples on the 2-D ensemble and 3-D U-Net performance is presented.

### 3.1 Comparison of 2-D, 2-D Ensemble, and 3-D U-Net Performance and Training Strategies

**Fig. 3:**
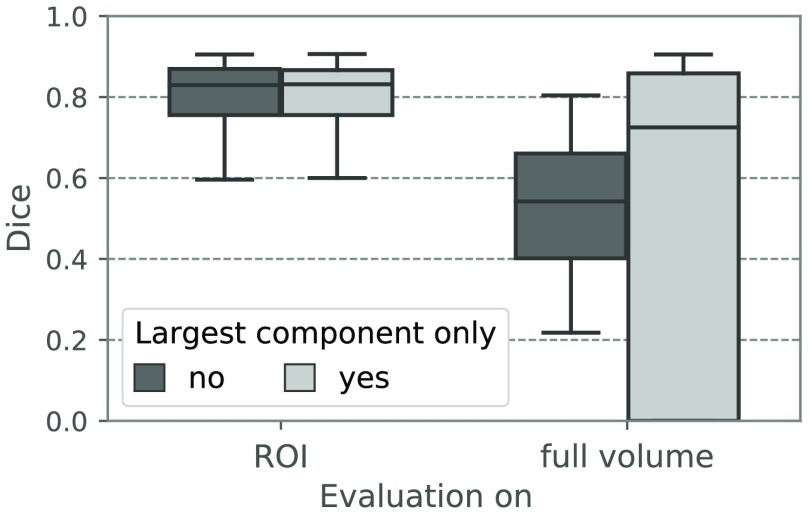
Dice coefficients of contours generated on the validation data by the axial 2-D U-Net when evaluated on an ROI versus the full volume. Dice scores were calculated once before and once after automatic selection of the largest CC in the binarized result mask.

Figure 3 shows the impact of the ROI-based training strategy onto the axial 2-D U-Net’s segmentation performance. The Dice coefficient was calculated on all 40 validation cases once on an ROI around the target structure (extracted the same way as during training) and once on the full volume. Additionally, it was computed before and after the postprocessing step of automatically selecting the largest CC. Within the ROI, the 2-D U-Net achieves a median Dice coefficient of 0.830 before and 0.831 after CC selection. The difference between the two median Dice scores is very small, as there are very few false positives next to the segmented parotid gland within the ROI. However, when evaluated on the full volume, the median Dice coefficient drops significantly to 0.542 before and 0.725 after CC selection, with 18 cases with a Dice coefficient of 0 for the latter. This means that within the ROI, the 2-D U-Net can on average accurately segment the parotid gland, but there are many outliers in distant body regions that are also large in volume. This could have been expected, as with the ROI-based training strategy, the U-Net never saw patches from outside the ROI.

**Fig. 4:**
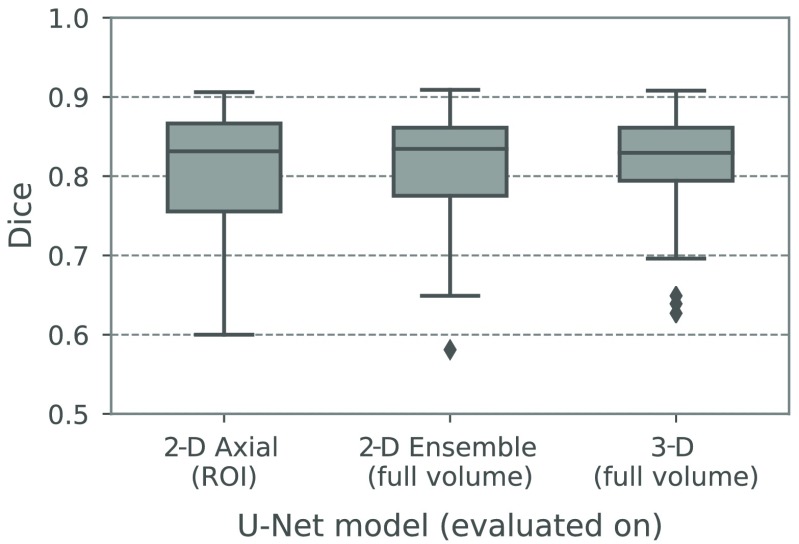
Dice coefficients of results generated by the axial 2-D U-Net (on an ROI only), the 2-D U-Net ensemble, and the 3-D U-Net on the validation data.

In Fig. 4, the 2-D U-Net performance is compared to the 2-D ensemble and the 3-D U-Net. With the idea to provide an automatic ROI detection in the future, the 2-D U-Net performance is shown on the ROI only as discussed before, whereas the other two neural networks models are evaluated on the full volume of all validation cases. All Dice coefficients in the plot were computed after the CC selection step. The median Dice coefficients of the 2-D ensemble (0.835) and the 3-D U-Net (0.830) are comparable to the 2-D U-Net (0.831) and the differences shown in Fig. 4 are not significant (p>0.05). The main observation is that both 2-D ensemble and 3-D U-Net segmentations contain only small false positives, which can be eliminated via CC selection so that the Dice coefficient on the full volume is comparable to that of the 2-D U-Net on the ROI. In the case of the 2-D ensemble, the combination of three individual predictions in orthogonal view directions can eliminate false positives of each single prediction. In the 3-D case, the patch-based training strategy helps to eliminate large false positives outside the ROI, as during the training, background patches from the whole training volumes are presented to the neural network.

**Fig. 5:**
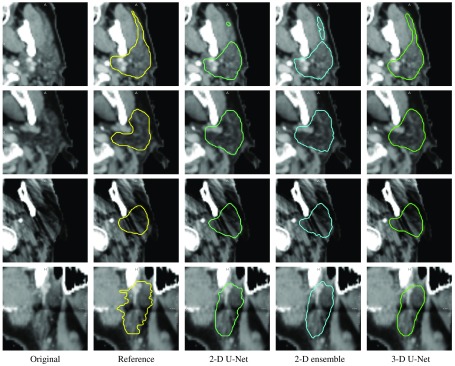
Reference contours and segmentation results by the axial 2-D U-Net, the 2-D U-Net ensemble, and the 3-D U-Net for a selection of validation cases (one case per row).

Figure 5 contains reference segmentations and deep learning results on selected exemplary validation cases that highlight typical observations. First of all, the segmentations by the three different neural networks are in general very similar, as also reflected in the Dice coefficients in Fig. 4. Most differences can be observed at the elongated lateral part of the parotid gland (first row), which is challenging for all neural networks. One common inconsistency between reference and autogenerated contour that can be observed in the second row is the inclusion of vessels at the medial part, which is consistent among all neural networks. This might be due to inconsistent annotations in the reference set. All trained neural networks are robust to dental metal artifacts (third row). In the exemplary case, they generate contours that even seem superior to the reference contours especially at the lateral anterior part. The fourth row shows contours of the third case in a sagittal plane. All neural networks, also the pure 2-D axial network, produce smooth contours in contrast to the reference segmentation, which often has an anatomically inconsistent shape in sagittal and coronal reformatted views. This might partially be due to resampling of the contours to the original image grid, which takes the segmentation from neighboring slices into account.

### 3.2 Comparison of Results on Validation and Independent Test Data

**Fig. 6:**
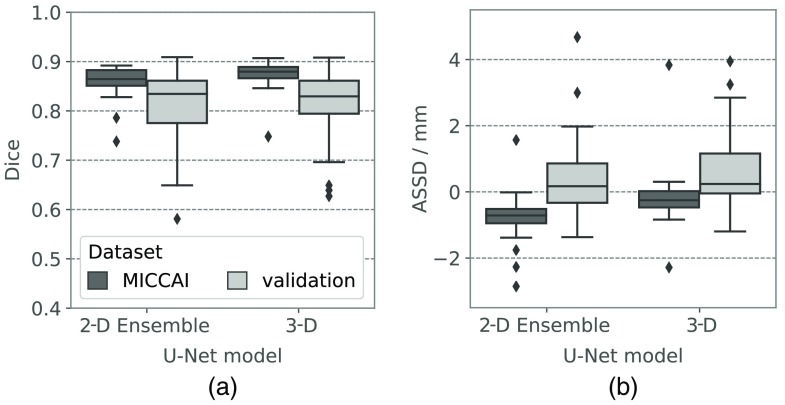
(a) Dice coefficients and (b) ASSD of segmentation results generated by the 2-D ensemble and the 3-D U-Net on the validation data and on the independent MICCAI test data.

**Fig. 7:**
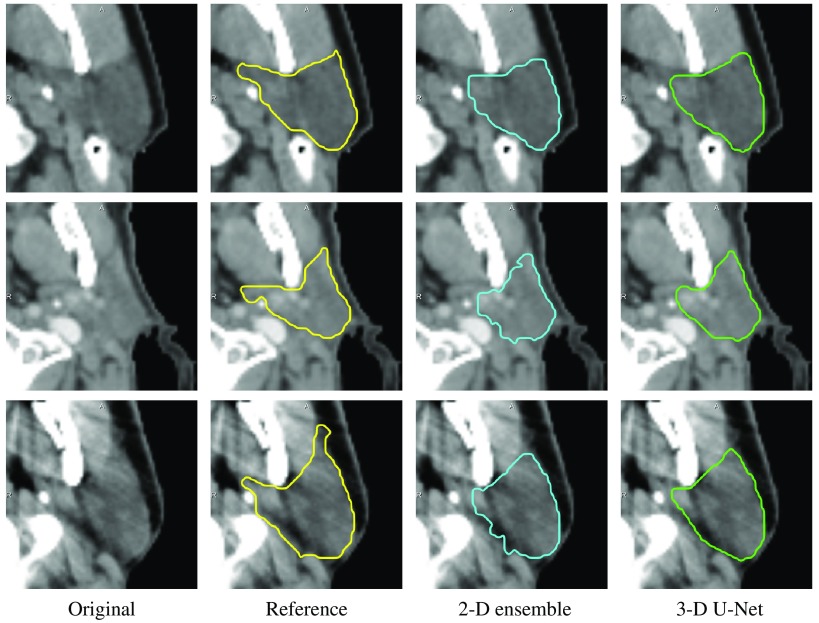
Reference contours and segmentation results by the 2-D U-Net ensemble and the 3-D U-Net for three MICCAI test cases (one case per row).

Both the 2-D ensemble and the 3-D U-Net were evaluated on the 15 test cases of the 2015 MICCAI challenge on head and neck autosegmentation. The axial 2-D U-Net was omitted due to the inability to compute the correct segmentation on the full volume after CC selection as seen in Sec. 3.1. Figure 6(a) shows the resulting Dice coefficients on the MICCAI test data compared to the results on the validation data from Sec. 3.1. The median Dice coefficient on the MICCAI data (0.865/0.880 for 2-D ensemble/3-D U-Net) is higher than on the validation data (0.835/0.830) and the variance is lower. This might be because uncurated data from clinical routine was used for validation (and training) that may contain errors that lead to lower Dice coefficients even for correct segmentations by the deep neural network. In Fig. 6(b), the average signed surface distance of the segmentation results to the reference is plotted. On the validation data, the median is close to zero for both 2-D ensemble (0.172 mm) and 3-D U-Net (0.239 mm), which means there is little bias toward under- or overestimation. The 2-D ensemble underestimates (negative ASSD) all but one MICCAI test case, the 3-D ensemble underestimates 21 of the 30 test cases. One common mistake of both neural network models is the underestimation of the medial part of the parotid gland that can be observed in Fig. 7. This might be due to the use of different contouring guidelines for the creation of the training versus MICCAI test contours, which leads to a systematic error during inference and Dice computation.

### 3.3 Comparison to Results of the 2015 MICCAI Challenge on Head and Neck Autosegmentation

**Fig. 8:**
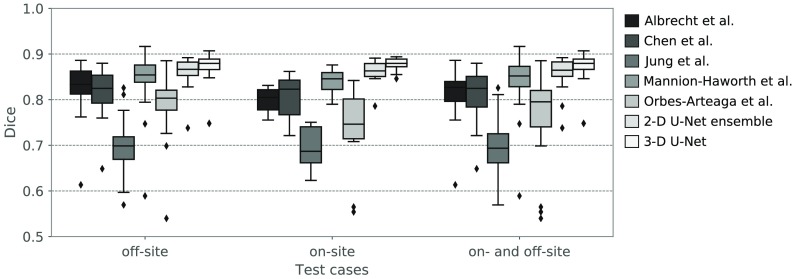
Dice coefficients of contours produced by the 2-D ensemble and the 3-D U-Net on the 2015 MICCAI challenge test data in comparison to the methods that competed in the challenge. For the challenge, the test data were split into off-site and on-site test cases, which is reflected in the plot.

In Fig. 8, the Dice coefficients computed from the segmentations by the 2-D ensemble and the 3-D U-Net are plotted together with the results of the MICCAI challenge on head and neck autosegmentation held in 2015. The original challenge results were published by Raudaschl et al.^13^ and kindly provided to us for comparison to our deep learning results. Results are plotted for the challenge’s on-site, off-site, and combined (on- and off-site) test cases separately, as some of the methods differ significantly for the on-site and off-site test cases. The two neural networks, however, yield similar quantitative results on each subset (see Table 1), which implies that the test cases in the two subsets are of similar difficulty. On the combined test set, the 3-D U-Net segmentations result in significantly (p<0.05) higher Dice coefficients than all methods in the challenge. For the 2-D ensemble, all differences are significant except for the comparison to Mannion-Haworth et al. (p=0.786). One has to keep in mind that in contrast to the methods in the challenge, our neural network models were trained on a completely independent training dataset. These results are promising as they demonstrate that deep learning methods can outperform other methods and are robust even when applied to independent data.

**Table 1:** Median Dice coefficients of the teams in the 2015 MICCAI challenge and the U-Net approaches for on-site, off-site, and combined test dataset.

Segmentation method/test cases	Off-site	On-site	All
Albrecht et al.^11^	0.833	0.805	0.827
Chen et al.^6^	0.825	0.823	0.825
Jung et al.^8^	0.699	0.687	0.694
Mannion-Haworth et al.^9^	0.854	0.846	0.852
Orbes-Arteaga et al.^10^	0.803	0.746	0.795
2-D U-Net ensemble	0.867	0.863	0.865
3-D U-Net	**0.880**	**0.880**	**0.880**

### 3.4 Impact of Training Data Quantity

**Fig. 9:**
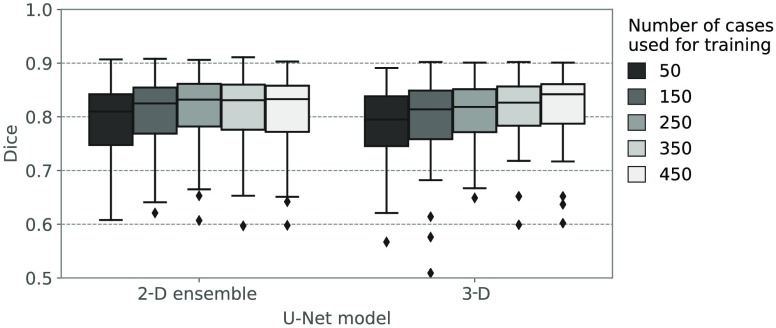
Dice coefficients of contours produced by the 2-D ensemble and the 3-D U-Net on the validation data when trained using an increasing number of training samples.

Figure 9 shows the results of the training of the 2-D ensemble and 3-D U-Net on increasing amounts of training data. Dice scores are reported on the validation data only. Even though the differences in the Dice coefficients on the validation data are small, the median Dice coefficient increases significantly (p<0.05) from 50 to 150 to 250 training cases and for both models. When adding more training cases, the median Dice coefficient for the 3-D U-Net still increases, but the differences are not significant for either neural network.

## 4 Discussion

While the results for all investigated neural network models were similar throughout our experiments, the 3-D U-Net seems most promising with respect to contour quality and also model simplicity compared to an ensemble approach with three distinct models. Moreover, the ensemble sometimes produces artifacts at the contour borders due to the majority voting, which will require further postprocessing such as smoothing. An important aspect to keep in mind when comparing the different approaches are the different numbers of parameters in the 2-D and 3-D U-Nets. With more parameters, the 3-D U-Net has a higher learning capacity than the 2-D U-Net and the ensemble, which may also partially explain the observed differences in performance. An option would be to use a deeper 2-D U-Net with one more resolution level, so that more parameters are available to the 2-D neural network. However, this would automatically increase the receptive field size of the 2-D U-Net compared to the 3-D U-Net (with respect to the axial plane), so that again no fair comparison could be granted.

In general, fair comparison of different neural network architectures with different numbers of parameters is difficult, in the sense that changing one parameter such as network depth directly impacts other network properties (such as receptive field). Hence, while comparing to other popular architectures different from the U-Net may yield interesting results, it would not be evident why one method outperforms the other and which conclusions to draw. Therefore, comparison of different architectures and training strategies as we have done in this work may give first directions toward which network architecture or sampling strategy should be investigated in more detail. The chosen parameters should then be optimized in a hyperparameter search, in which only minimal changes are made to the training. That way, the impact of single parameters can be analyzed in a fair comparison. For example, future work could focus on binary versus nonbinary segmentation with the 3-D U-Net, such as simultaneous segmentation of right and left parotid gland or inclusion of further OAR in the head and neck region. Such a neural network would have only slightly more parameters due to the increased number of output channels but produce multiple segmentations in only slightly increased amount of time. It should then be investigated how the generalization capacity of a neural network changes, when it has to segment several distinct anatomical structures instead of a single one.

Furthermore, as the results produced by different neural networks can be similar as observed in Fig. 5, it would be interesting to compute local, application-specific distance measures instead of global general ones such as Dice score or surface distances. For example, one could then focus on the lateral elongated part of the parotid gland and assess model robustness with respect to this specific anatomical feature.

A limitation of quantitative results on the validation data as seen in Secs. 3.1 and 3.4 is the fact that they are not completely reliable as uncurated reference contours from clinical routine were used. The effect of this could be seen in Fig. 6, where the variance of the Dice scores on the uncurated validation data was much higher than on the curated test data. Assuming that the validation cases are not significantly harder to segment than the test cases, the increased variance may likely stem from the uncertainty in the reference masks. For future work, having a subset of the data for validation with well-curated reference contours would be highly desirable for quantitative evaluation. Still, the difference in results between the neural networks indicate that a patch-based training strategy with controlled batch composition is to be preferred over an ROI sampling strategy with respect to false-positive reduction. An automatic ROI detection, e.g., using a smaller neural network working on a coarse resolution would, however, still be desirable in order to speed up inference.

The use of clinical quality reference contours in Sec. 3.4 also poses the question whether the amount of available data is sufficient for the problem at hand as one might read from the plots. Another interpretation is that with the currently available clinical reference quality, no better results can be achieved no matter how much more data of similar quality is added to the training. We have conducted first experiments on training with curated versus clinical quality reference contours.^40^ Our results suggest that the difference in segmentation performance between training on a large dataset of clinical quality references versus a smaller dataset of curated quality references is small. After this rather technical study, an important further step will be to do a clinical qualitative evaluation of the clinical acceptance of the contours generated using deep learning. It needs to be investigated whether contours derived from clinical uncurated quality data via deep learning can fulfill clinical contouring guidelines. Another train of thought is that deeper networks with more parameters may also be able to integrate more information and further improve the segmentation performance with more samples. However, a fair comparison to a deeper neural network would be difficult due to the problems already discussed.

Finally, the results of Sec. 3.3 demonstrate the generalization potential of deep learning methods for autocontouring of CT data from different sites. We suppose that this is primarily due to the availability of the image data in well-calibrated Hounsfield units. Autosegmentation of MR planning images or cone-beam CT images for adaptive radiotherapy could be more challenging with respect to homogeneous datasets and generalization. In that case, training of neural networks for a specific site and scanner may be necessary. Moreover, the deep learning methods show a higher robustness or lower variance in performance compared to the atlas- and model-based methods in the segmentation challenge. This may be because the learned features can represent a large anatomical variety without prior assumptions and have additionally been trained on a larger dataset than the methods in the challenge.

## 5 Conclusion

We have presented results on parotid gland segmentation using deep learning, including an analysis of the amount of training data. Using the U-Net architecture and using uncurated reference segmentations from clinical routine for training, we can achieve results on a publicly available test set that are significantly better than those by several model- and atlas-based methods. This demonstrates the high potential of deep learning-based autosegmentation methods for radiotherapy planning, where manual or semiautomatic contouring is still a bottleneck in the workflow.
